# Antiseptics at Guy's Hospital

**Published:** 1898-10-22

**Authors:** 


### ANTISEPTICS AT GUY'S HOSPITAL.

A heavy responsibility rests upon the editor of every paper, and when, as is the case with the *Guy's Hospital Gazette*, a paper represents a great hospital and a great school, although it is the editor's bounden duty to lay bare and aid in the reform of any abuse which exists, it is equally his duty not to print anything which shall suggest evil if none is present. These being our views as to editorial duty, we confess to a feeling of some surprise at a paragraph which occurs in the *Guy's Hospital Gazette* for October 8th in the course of a review of a work by Dr. Macnaughton-Jones on Asepsis and Antisepsis in Abdominal Surgery and Gynæcology. The paragraph, which is evidently "writ sarcastic," and with the intention of having a slap at the gynæcologists of Guy's Hospital, runs as follows:—

"Accustomed as the mind of the reviewer is to new sensations, on this occasion it was filled with wonderment and surprise. Nor is the cause far to seek. It seemed incredible that in London in the year 1898 anyone should be found willing to waste his time in writing on asepsis for the especial benefit of gynæcologists—who, as everybody knows, are in the front rank among the asepticians of the world. Here, in Guy's, for instance, is not Queen Victoria ward about to be opened? Have not the gynæcologists been repeatedly observed (by impartial witnesses) dipping their hands in perchloride of mercury (1 in 2,000) for at least fifteen seconds before proceeding to an abdominal section? Have not those members of the nursing staff who assist at gynæcological operations been instructed that instruments, sutures, &c., which have been dropped on the floor, or into the sawdust-tray, should be momentarily immersed in lysol before being handed to the operator? We emphatically ask, what more is necessary?"

We would ask, then, Are the deficiencies in the antiseptic precautions taken by the gynæcologists of Guy's Hospital so great, and are they matters of such common knowledge, as to justify the publication of such a paragraph in the hospital's own *Gazette?* If not, this paragraph is surely badly inspired. On the other hand, if such things are—if it is true that a fifteen seconds' immersion in 1 in 2,000 perchloride solution is looked upon as enough for the hands, and if it is true that, when instruments, sutures, &c., have been dropped on the floor, nurses are allowed to hand them to the operator after being "momentarily immersed in lysol"—then we ask, Is the *Guy's Hospital Gazette* fulfilling the proper function of journalism in remaining silent on such matters, and merely relieving its feelings by a sarcastic reference to them in its review columns?

